# Preparation for the challenge of heavily mutated Omicron variant

**DOI:** 10.1002/ctm2.679

**Published:** 2021-12-12

**Authors:** Yuanlin Song, Fujita Masaki

**Affiliations:** ^1^ Department of Pulmonary Medicine Zhongshan Hospital Fudan University Shanghai China; ^2^ Department of Respiratory Medicine Fukuoka University Hospital Fukuoka Japan

The Omicron variant of SARS‐CoV‐2, also called B.l.1.1.529 mutant, has been considered as the most ferocious new crown variant so far.^1^ The first confirmed B.1.1.529 virus was collected on 9 November 2021. After the first report, infections of B.1.1.52 increased steeply in subsequent weeks. It was first reported to World Health Organization from South Africa on 24 November 2021 and was designated as a variant of concern, named Omicron. For viruses adapting to their human hosts, such as SARS‐CoV‐2,^2^ genetic evolution continues with developing mutations over time, which results in mutant variants that may have different characteristics than its ancestral strains. The receptor‐binding domain (RBD) in the spike protein of severe acute respiratory syndrome coronavirus 2 (SARS‐CoV‐2) binds to the angiotensin‐converting enzyme‐2 on the host cell surface with high affinity, which is the origin of several SARS‐CoV‐2 variants.^3^ Many changes had little to no impact on the virus’ properties. However, some adaptive mutations in the SARS‐CoV‐2 genome which altered its pathogenic potential would increase the transmission ability or disease severity.

There were 32 mutations in the Omicron variant, which is the largest number of Spike protein mutations among mutant strains.^4^ The number of mutations was twice the Delta variant. Ten mutations were found on the RBD of the Omicron variant, while there were only two in Delta variants. These mutations of the spike proteins had been found in variants such as Delta and Alpha and were linked to increased transmissibility or immunity evading.^5^, ^6^ For example, the N501Y mutation of the Omicron variant increased the affinity of the spike protein to ACE‐2 receptors, enhancing viral attachment and subsequent entry into host cells.^7^ Moreover, the Omicron variant mutated at E484 in RBD of the spike protein, which avoided human immune responses.^8^ The variant was also mutated at D614G mutation which might increase transmission and stability of viral replication in human lung epithelial cells.^9^ Furthermore, the N protein of the Omicron variant has R203K/G204R mutations which are related to the Alpha variant (B.1.1.7). R203K/G204R mutations would increase adaptability by the virus evolution analysis using the computation biology analysis.^4^ In addition, the Omicron variant also had several mutations that did not report previously, whether they would enhance its infectivity and the disease severity, their roles should be clarified. The Omicron variant epidemiological characteristics, transmissibility and immune escape would take time to be comprehensively identified.

However, the unvaccinated population were most vulnerable to the Omicron variant.^10^ The number of 120 000 people vaccinated per day were lower than the target of 300 000 per day by the plan of the South African government. About 30%–40% of South African adults had been vaccinated, while 65% of young patients with moderate or severe COVID‐19 had not been vaccinated and most of them had only one shot of vaccine. Vaccines might still provide good protection for SARS‐CoV‐2 variants. Most vaccines were based on spike proteins of SARS‐CoV‐2 to activate immune cells, so the impact of the Omicron variant on existing vaccines should be evaluated as soon as possible. Two or three shots of most vaccines should be needed to stimulate acquired immunity. The emergence of antibodies against SARS‐CoV‐2 which produced protective antibodies and induced memory cells may be activated in response to subsequent exposure to SARS‐CoV‐2 and its variants.^11^

Recently, a novel COVID‐19 oral antiviral candidate PF‐07321332 (Paxlovid) reduced the risk of hospitalization or death by 89% compared to placebo in an interim analysis of phase II/III study by Pfizer.^12^ PF‐07321332 was designed to block the activity of the SARS‐CoV‐2‐3CL protease, an enzyme that participated in virus replication. It inhibited viral replication at a stage known as proteolysis, which occurs before viral RNA replication.^13^ Co‐administration with a low dose of ritonavir might slow down the metabolism or breakdown of PF‐07321332 to remain active and maintain high concentration for a longer time to combat the virus. For individuals, a variety of measures are still recommended to reduce the risk of virus exposure, including maintaining social distance, wearing masks, washing hands, avoiding crowded places, room ventilation, and timely vaccination. These proven measures are particularly important at the time.
